# Mapping the Prion Protein Distribution in Marsupials: Insights from Comparing Opossum with Mouse CNS

**DOI:** 10.1371/journal.pone.0050370

**Published:** 2012-11-29

**Authors:** Ilaria Poggiolini, Giuseppe Legname

**Affiliations:** 1 Department of Neuroscience, Laboratory of Prion Biology, Scuola Internazionale Superiore di Studi Avanzati (SISSA), Trieste, Italy; 2 ELETTRA Laboratory, Sincrotrone Trieste S.C.p.A., Basovizza, Trieste, Italy; Creighton University, United States of America

## Abstract

The cellular form of the prion protein (PrP^C^) is a sialoglycoprotein widely expressed in the central nervous system (CNS) of mammalian species during neurodevelopment and in adulthood. The location of the protein in the CNS may play a role in the susceptibility of a species to fatal prion diseases, which are also known as the transmissible spongiform encephalopathies (TSEs). To date, little is known about PrP^C^ distribution in marsupial mammals, for which no naturally occurring prion diseases have been reported. To extend our understanding of varying PrP^C^ expression profiles in different mammals we carried out a detailed expression analysis of PrP^C^ distribution along the neurodevelopment of the metatherian South American short-tailed opossum (*Monodelphis domestica*). We detected lower levels of PrP^C^ in white matter fiber bundles of opossum CNS compared to mouse CNS. This result is consistent with a possible role for PrP^C^ in the distinct neurodevelopment and neurocircuitry found in marsupials compared to other mammalian species.

## Introduction

The cellular form of the prion protein (PrP^C^) is a cell-surface glycosylphosphatidylinositol-anchored glycopolypeptide abundantly expressed in the central nervous system (CNS), with expression levels varying among different cell types and brain regions [1]. The distribution pattern of PrP^C^ has already been investigated in deep detail in the CNS of several placental mammalian organisms, including mouse (Mo) [2]–[6], hamster [7], cattle [8], sheep [9] and primates [10], [11]. Additional studies, along the same lines of research, have reported the pattern of PrP^C^ distribution also in avian [12] and fish [13]. The earliest expression of the protein in mammals has been observed in the hippocampus, thalamus and hypothalamus and the highest levels of PrP^C^ expression have been noted in specific white matter fiber tracts [6].

**Figure 1:**
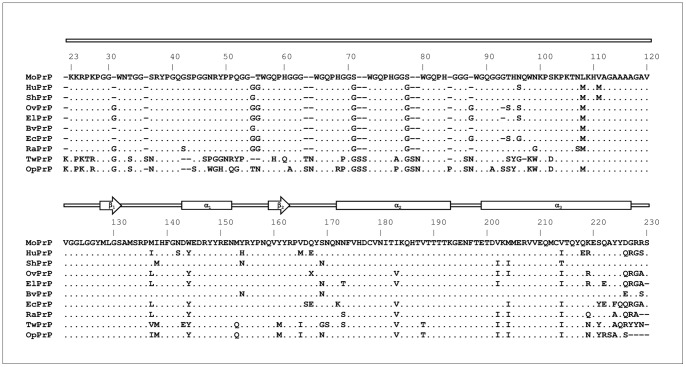
Comparison of amino acid sequences and secondary full-length prion protein structure of selected mammalian species: mouse (MoPrP; *Mus musculus*, GenBank accession number: AAA39997), human (HuPrP; *Homo sapiens*, AAA60182), Syrian hamster (ShPrP; *Mesocricetus auratus*, AAA37091), sheep (OvPrP; *Ovis aries*, ABC61639), elk (ElPrP; *Cervus elaphus nelsoni*, AAB94788), bank vole (BvPrP; *Clethrionomys glareolus*, AAL57231), horse (EcPrP; *Equus caballus*, ABL86003), rabbit (RaPrP; *Oryctolagus cuniculus*, AAD01554), tammar wallaby (TwPrP; *Macropus eugenii*, AAT68002) and opossum (OpPrP; *Monodelphis domestica*, CBY05848).

Structurally, mature PrP^C^ expressed by a wide variety of mammalian species shares a similar fold: while the N-terminus is largely unstructured, the C-terminus possesses well-defined secondary and tertiary structures [14], [15]. The N-terminus features an evolutionarily conserved motif denoted as the octapeptide-repeat region (residues from 51 to 90 in Mo numbering, Figure 1). The octapeptide-repeat region is able to coordinate the binding of copper ions, thus implicating a possible role of PrP^C^ in copper homeostasis [16].

The sequence identity of PrP^C^ among mammals suggests an important physiological role [17]. However, the function of the protein has not been fully clarified, mostly due to the fact that PrP^C^-null mice (*Prnp* ^0/0^) do not show remarkable phenotypic abnormalities [18], [19]. Putative PrP^C^ functions are based on its localization. In particular, the highest expression of the protein in the hippocampus and, within this brain region, in the *stratum lacunosum-moleculare*, suggests a role for PrP^C^ in synaptic structure, function and maintenance [6]. Additionally, the large number of PrP^C^-interacting molecules identified thus far [20] implies that PrP^C^ may be a dynamic cell surface platform for the assembly of signaling modules.

Defining the function of PrP^C^ is also a prerequisite for understanding TSEs, or prion diseases, as they are attributed to the posttranslational conversion of PrP^C^ into a misfolded, pathogenic form denoted prion or PrP^Sc^ [21]. This group of rare neurodegenerative maladies, affecting humans and animals alike, can be sporadic, genetic or iatrogenic. They include Creutzfeldt-Jakob disease (CJD), fatal familial insomnia and Gerstmann-Sträussler-Scheinker syndrome in humans, scrapie in sheep and goats, bovine spongiform encephalopathy in cattle, and chronic wasting disease in cervids.

A still controversial aspect in TSEs is the different ability of prions to infect some mammalian species and not others. So far no naturally occurring TSEs have been reported in rabbit, horse or any marsupial species. A possible explanation for this argues that the PrP^C^ primary sequence, together with local structural variations within the C-terminus globular domain, might account for prion resistance in different mammals [22]. However, little is known about the regional distribution of PrP^C^ in the CNS of mammalian species that seem resistant to TSEs. Differences in PrP^C^ expression in mammalian species, for which no naturally occurring TSEs occur, may shed light on different susceptibility to these maladies.

To gain insights into this neglected issue we analyzed PrP^C^ distribution along the neurodevelopment of the metatherian mammal South American short-tailed opossum (*Monodelphis domestica*) (hereafter Op). This animal model is used in developmental studies mainly because of the rudimental stage of development of the newborn pups, which resemble 11- or 12-day Mo embryos [23], [24]. In the newborn Op pup the CNS is still at an embryonic stage [23], because its development is completed during postnatal life.

The Op genome sequencing has provided an important tool for comparison with Eutherians, such as human and Mo, and has contributed to our knowledge about the evolution of Amniota [25], [26]. Because of its evolutionary position between avian and eutherian genomes, Op represents an invaluable model for evolutionary comparison. Ultimately, its small size, ease of care and the non-seasonal breeding make Op a suitable laboratory animal model [27], [28].

The assignment and characterization of Op prion protein (PrP) gene has revealed that OpPrP and MoPrP share approximately 70% sequence identity [29]. Sequence variations are most prominently localized on the N-terminus copper binding sites region: while MoPrP contains one nonapeptide, and four octarepeats of identical sequence, OpPrP features five different decarepeats, which are able to bind copper ions [30], [31]. Additionally, the region from residue 91 to 110 (in MoPrP numbering), which also binds copper, is less conserved in OpPrP (Figure 1).

In this work, the expression profile of OpPrP was characterized at different postnatal developmental stages of Op CNS using Western blotting and histoblot techniques. To compare OpPrP and MoPrP distribution in CNS, we examined the expression of PrP^C^ in postnatal 30-day-old (P30) mice – which resemble young adult Op in the overall development – under the same experimental conditions.

The most striking difference between the two mammals concerned the lower PrP^C^ detection in the Op white matter structures. The different organization pattern observed might offer insights into the role of PrP^C^ in neurodevelopment and in neurocircuitry formation in Op and other mammals [32]. Ultimately, it might also expand our current knowledge of PrP^C^ function in mammals.

## Materials and Methods

### Animals

All experiments were carried out in accordance with European regulations [European Community Council Directive, November 24, 1986 (86/609/EEC)] and were approved by the local veterinary service authority. FVB wild-type, and FVB *Prnp* ^0/0^ mice [33] were used in these experiments. Animals were obtained from the colony maintained at the animal house facility of the University of Trieste, Italy. Animals were staged by systematic daily inspection of the colony for newborn litters. P0 corresponds to the day of birth [34]. Each experiment was performed at least in triplicate. Mice and Op pups were decapitated. Mice (at P30) and Op adults (at P45, P50, and P75) were killed by cervical dislocation. For histoblotting, brains were rapidly harvested, immediately covered in powdered dry ice and included in the embedding medium OCT (Optimal Cutting Temperature).

### Histology

CNS specimens were fixed in 4% paraformaldehyde-PBS overnight at 4°C, cryoprotected in 30% sucrose/PBS and cut coronally at 20 μm. Cryosections were mounted on Fischer SuperFrost Plus slides and subsequently processed for histology.

### Histoblots

The histoblot technique was performed according to the protocol described by Taraboulos et al. [35] with a few modifications. Briefly, uncoated microscope slides (Menzel-Glaser, Madison, WI) carrying 20 µm-thick brain serial coronal sections were pressed onto a nitrocellulose membrane wetted in lysis buffer (0.5% sodium deoxycholate, 0.5% Nonidet P-40, 100 mM NaCl, 10 mM EDTA, 10 mM Tris-HCl, pH 8.0), incubated for one hour at room temperature in 0.1 M NaOH and rinsed 3 times for 1 minute in TBST 1X (50 mM Tris-HCl, 150 mM NaCl, 0.1% Tween20, pH 7.4). Blots were blocked for 90 minutes in 5% non-fat dry milk-TBST 1X. They were incubated overnight at 4°C with the primary antibody anti-PrP^C^ humanized Fab D18 [36] purchased from InPro Biotechnology (South San Francisco, CA; ABR-0D18) and used at a final concentration of 1 μg/mL. This antibody shows high affinity for the region encompassing residues 133 to 152 (in Mo numbering), which is highly conserved in different mammals (Figure 1). Membranes were extensively washed in TBST 1X and incubated for one hour with secondary antibody diluted in blocking mix. The signal was achieved using SIGMAFAST^TM^ 3,3′-Diaminobenzidine tablets (Sigma) according to the protocols of the supplier. All data are representative of at least three independent experiments.

### Nissl staining

Twenty-micrometer fixed frozen cryostat sections, mounted on slides, were air-dried for 60 minutes, stained in 0.1% cressyl violet (Sigma) at 40°C for 7 minutes and then rinsed in distilled water. Slides were soaked in 95% ethyl alcohol for 5 minutes and dehydrated in 100% alcohol for 5 minutes. Before mounting on glass slides (Sigma) with resin medium (Eukitt, Bio-Optica) slides were cleared twice in xylene for 5 minutes.

### Western blotting analysis

Total brains or different brain regions were dissected using a stereomicroscope (Nikon SMZ 800) and immediately frozen in liquid nitrogen. Tissues were homogenized in RIPA buffer (150 mM NaCl, NP-40 1%, sodium deoxycholate 0.5%, SDS 0.1%, 50 mM Tris, pH 8.0) with Glass/Teflon Potter Elvehjem homogenizers and spun at 1000 g at 4°C for 5 minutes. The total protein amount was determined using the BCA Protein Assay Kit (Thermo Scientific Pierce). Fifty µg of total protein was then electrophoresed through 10%–SDS polyacrylamide gels and transferred to nitrocellulose membranes. Membranes were probed with monoclonal antibody Fab D18 and developed by enhanced chemiluminescence (Amersham ECL Western Blotting Systems, GE Healthcare). Band intensity was quantified using the UVI Soft software (UVITEC, Cambridge).

## Results

### PrP^C^ expression in the Op brain is developmentally regulated

**Figure 2:**
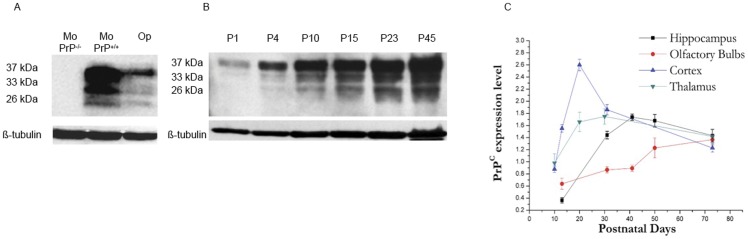
Confirmation of antibody specificity and developmental expression of PrP^C^ in opossum CNS. (A) Western blotting of Op, MoPrP^+/+^ and MoPrP^−/−^ whole brain homogenates confirmed the specificity of the PrP^C^ signal. The blot was then reprobed with β-tubulin antibody to demonstrate equal loading of samples (50 µg per lane). (B) Western blotting analysis of equal amounts of total brain homogenate (50 µg per lane) at different developmental stages showed a major electrophoretic band at 37 kDa, which corresponds to the diglycosylated form of the protein. β-tubulin was used as loading control. (C) Western blotting of indicated brain regions at different developmental stages showed a relevant change in PrP^C^ expression during postnatal development. Each data point represents the mean absorbance ±SEM of 3 females from different litters. All the absorbance values were normalized against β-actin.

PrP^C^ protein extracts from adult knockout PrP (MoPrP^−/−^), wild-type PrP (MoPrP^+/+^) mice and adult Op were compared using Western blotting (Figure 2A). The expected di- (∼37 kDa), mono- (∼30 kDa) and un- (∼27 kDa) glycosylated forms were detected by Fab D18 monoclonal antibody both in MoPrP^+/+^ and Op lanes. The absence of signal in the lane loaded with MoPrP^−/−^ sample showed the specificity of the antibody. Although all the lanes were loaded with the same amount of total protein, the lower intensity of Op PrP signal compared to MoPrP^+/+^ signal might be due to a lower affinity of the antibody for the Op PrP than for MoPrP^+/+^. Alternatively, these results might indicate a lower PrP^C^ expression in adult Op than in Mo. Figure 2B shows the PrP^C^ pattern observed by immunoblotting from P1 to P45. A predominance of the diglycosylated form of the protein at ∼37 kDa and of the monoglycosylated form of the protein at ∼30 kDa was observed. A minor band corresponding to the non-glycosylated form of the protein was detected at ∼27 kDa. This expression pattern resembles those observed in eutherian Syrian hamster (SHa) and Mo brains [37].

The PrP^C^ expression from P10 to P70 was also evaluated in the thalamus, olfactory bulbs, cortex and hippocampus (Figure 2C). In all these regions an increase in PrP^C^ expression was observed until P50. In adulthood the expression of PrP^C^ decreased slightly or remained at plateau. Like in SHa [7], a tendency to an increase in PrP^C^ signal was observed in the olfactory bulbs at P75.

### Regional expression of PrP^C^

**Figure 3:**
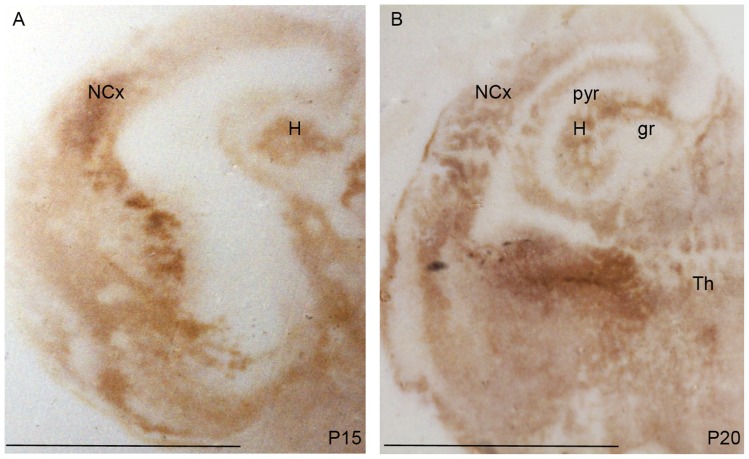
PrP^C^ expression in histoblots of P15 and P20 opossum brains. (A–B) In coronal sections of P15 and P20 a PrP^C^ signal was detected in the thalamus (Th), in the neocortex (NCx) and in the hippocampus (H). The pyramidal cell layer (pyr) and the granule cell layer (gr) of the hippocampus were not stained by PrP^C^ (Bars: A–B 4 mm).

**Figure 4:**
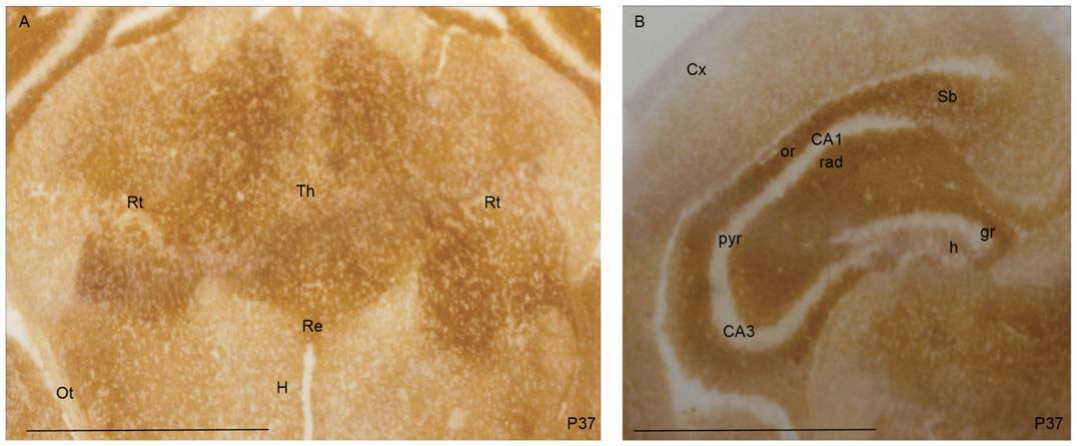
PrP^C^ distribution in a P37 opossum brain. (A–B) Coronal sections in the caudal diencephalon from a P37 opossum were stained for PrP^C^ in the thalamus (Th) and in the parenchyma of the hippocampus. (A) In the ventral thalamus, a strong IR was observed in the reticular nucleus (Rt) and in the nucleus reuniens (Re). No IR was observed in the optic tract (Ot). In the hypothalamic area (H) the immunoreactivity was lower than in the thalamus. (B) A strong PrP^C^ signal was observed in the *stratum oriens* (or) and in the *stratum radiatum* (rad) of the hippocampus. A low PrP^C^ immunoreactivity was observed in the hilar region (h), while the pyramidal (pyr) and granule (gr) cell layers of the CA1-CA3 and DG were devoid of immunostaining (Bars: 1 mm).

Since PrP^C^ was not detected by immunofluorescence staining performed following well-established protocols described in the literature, we investigated the regional distribution of PrP^C^ in the Op brain at P15, P20, P37 and P70 by histoblot (see Materials and Methods) [35]. After the completion of cortico-cerebral neurogenesis [34], at P15 and at P20 strong PrP^C^ immunoreactivity was detected in the hippocampus, in the thalamus and in the neocortex. In the hippocampus, a signal was observed in the parenchyma, but not in the pyramidal layer of the Ammon's Horn (CA1-CA3) nor in the granule cell layer of the dentate gyrus (DG) (Figure 3A–B). At P37 (Figure 4A and B) a dense PrP^C^ signal was identified in gray matter structures such as thalamus, cortex and hippocampus. In the latter, PrP^C^ immunostaining was deep in the *stratum radiatum* and in the *stratum oriens*. As observed at P20, the signal was virtually absent in the pyramidal cells of the CA and in the granule cells of the DG. In the hilar region, immunoreactivity (IR) was minimal. IR was observed around the dorsal and the lateral parts of the thalamus (Figure 4A), encompassing structures involved in the communication between cortex and thalamus [38].

**Figure 5:**
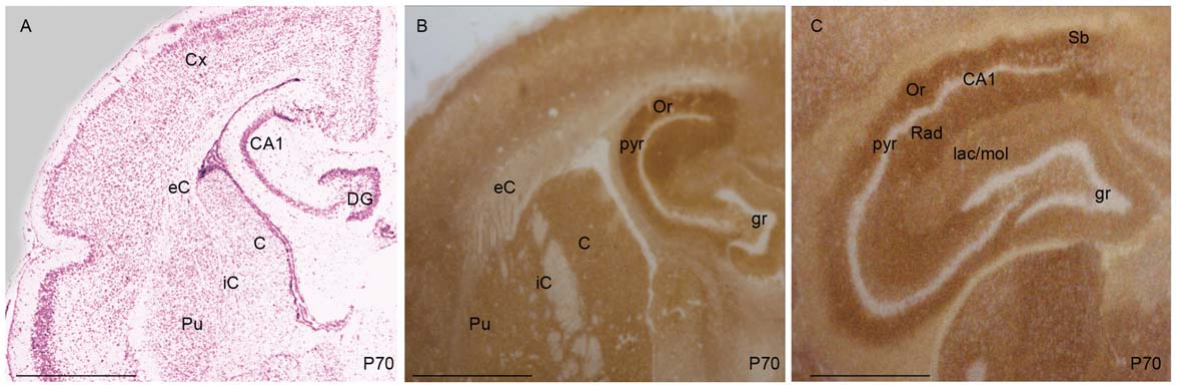
Coronal sections of opossum brain at P70 (A–C). In (A) the coronal section was Nissl-stained. In the adult opossum (B) a strong signal was predominantly present in the hippocampus. Marginal signal was also detected in the caudate nucleus (C), in the internal capsule (iC) and in the putamen (Pu). A very residual signal was observed in the external capsule (eC). In (C) the specific distribution of PrP^C^ in the different hippocampal layers was analyzed. Staining in the *lacunosum* and *moleculare* (lac/mol) was lower than in the *oriens* (Or) and the *radiatum* (Rad). PrP^C^ immunoreactivity was also observed in the *subiculum* (Sb) the main output of the hippocampus. (Bars: 0.5 mm).

The PrP^C^ signal was also evaluated at P70, after the time of weaning [23]. A low IR was observed in white matter areas such as the internal and external capsules (Figure 5B). As observed at P37, in the P70 hippocampus the strongest IR was present in the *oriens* and *radiatum* strata. A less intense signal was detected in the *stratum lacunosum-moleculare* (Figure 5C). The expression pattern profile observed in adult Op hippocampus is similar to that in SHa [7].

### PrP^C^ immunolocalization in Mo brain coronal sections

**Figure 6:**
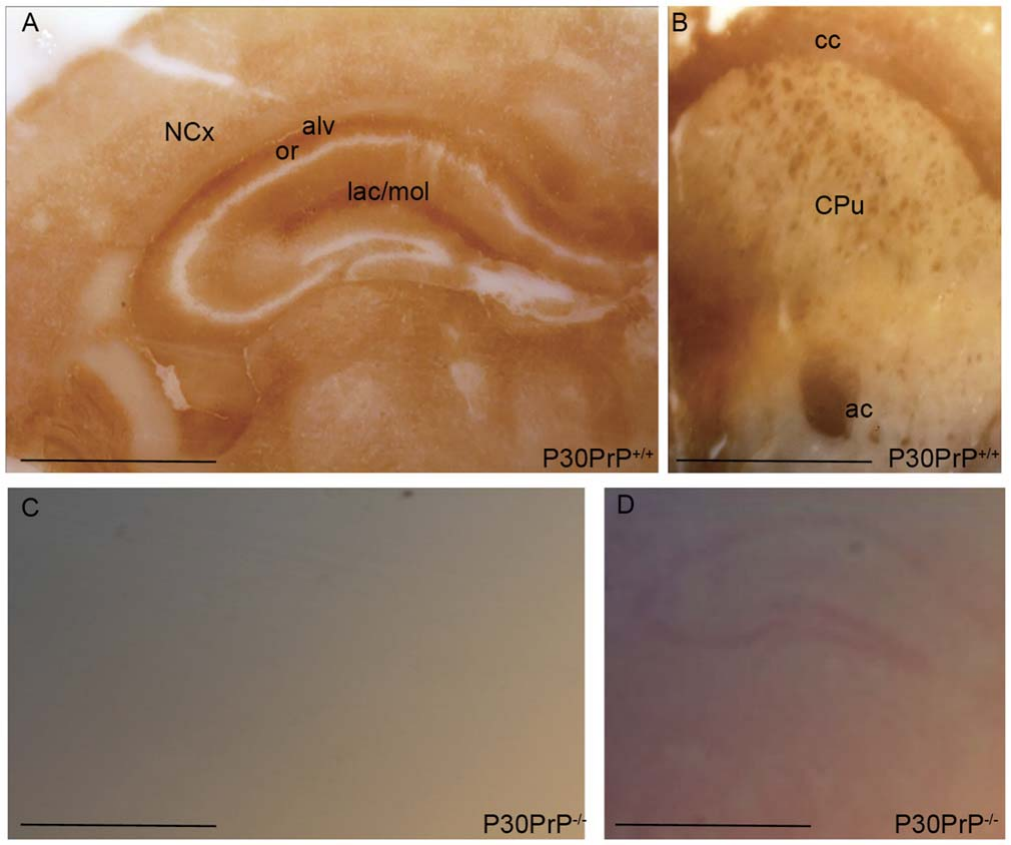
Localization of PrP^C^ in P30 MoPrP^+/+^ brain and control of signal specificity. (A) At P30 a well-defined signal was present in the hippocampal *stratum lacunosum-moleculare* layer (lac/mol) and in the alveus (alv) lying just deep to the *stratum oriens* layer (or). (B) In the septum-caudatum, PrP^C^ signal was detected predominantly in white matter fiber bundles, such as the anterior commissure (ac) and the *corpus callosum* (cc). The dark dots observed in the caudate-putamen (CPu) are fiber fascicles cut on end. (C) At P30, the lack of IR in MoPrP^−/−^ coronal section confirmed the signal specificity. (D) The presence of the brain section on the nitrocellulose membrane was confirmed by Ponceau staining. (Bars: A–D 0.5 mm).

Histoblots of P30 MoPrP^+/+^ brain coronal sections were immunostained to measure differences in PrP^C^ localization between Op and Mo (Figure 6A and B). The lack of IR in P30 MoPrP^−/−^ coronal sections (Figure 6C and D) confirmed the specificity of the D18 signal. Ponceau staining was performed to ensure the presence of the section on the nitrocellulose membrane.

The pattern of PrP^C^ distribution in P30 MoPrP^+/+^ was detected in many structures throughout the brain (Figure 6A and B). At P30 strong IR was found in the alveus, a thalamo-limbic structure of fornix fibers surrounding the *stratum oriens* that contains the axons of pyramidal neurons. As previously reported [6], a well-defined PrP^C^ signal was present in the *stratum lacunosum-moleculare* (Figure 6A). Strong labeling of the white matter fiber bundles was particularly evident at the level of the *corpus callosum* – the major interhemispheric fiber bundle in eutherians – and in the anterior commissure, also involved in interhemispheric communication [39]. Within the limbic system, a signal was detected in the hippocampal fimbria, in its continuation, the fornix and in the hippocampus. In the neocortex (NCx), staining was detected in a region adjacent to the ependymal layer.

## Discussion

Over the last twenty years, the expression of PrP^C^ in the CNS of placental mammals, such as Mo and SHa, has been intensively investigated. Here we described a restricted PrP^C^ expression during Op brain development. Our data appear to corroborate current evidence of a developmentally regulated expression of PrP^C^ in all mammals. The direct comparison between Op and MoPrP^C^ expression in CNS showed striking differences in distinct brain regions, such as white matter structures and hippocampus, thus suggesting possible functional implications for the role of PrP^C^ in marsupials.

### Technical remarks about PrP^C^ detection in Op brain

The routine histological techniques might not be sensitive enough to map PrP^C^ expression in the Op brain. We tested different immunofluorescence protocols in combination with several monoclonal antibodies, but none of them appeared to work (data not shown). We speculated that these technical difficulties experienced with the traditional immunohistochemical staining techniques might be due to a weak antibody affinity for OpPrP, possibly ascribable to epitope masking as a result of a different membrane environment. Alternatively, PrP^C^ signal might be masked by another molecule, which could make the binding of the antibody to the antigene inaccessible.

To overcome these difficulties we decided to use the immunohistoblot technique described by Taraboulos et al. [35] to map the regional distribution of PrP^Sc^ in the brain of diseased SHa. The use of 0.1 M sodium hydroxide enhanced the binding of PrP antibodies [40] thus allowing for the detection of a clear PrP^C^ signal in the cryostat sections of the freshly frozen Op brain tissues in this study.

### Comparison of PrP^C^ distribution between marsupials and placental mammals

Our results showed that from the day of birth (P1) up to adulthood (P75) PrP^C^ was detectable by Western blotting in whole brain homogenates (Figure 2B) with the strongest PrP^C^ signal in the uppermost diglycosylated band (∼37 kDa) and the weakest signal in the lowest non-glycosylated PrP^C^ band (∼26 kDa). A change in PrP^C^ relative abundance was observed during Op brain development, corroborating previous evidence of a developmentally regulated expression of PrP^C^. In the different brain regions under consideration, PrP^C^ levels either remained at plateau or decreased slightly in adulthood (Figure 2C). Interestingly, after the time of weaning a tendency to an increase in PrP expression was observed in the olfactory bulbs. As postulated for placental mammals [7] this finding might be related to ongoing plasticity of the olfactory bulbs also in marsupials. However, no evidence is available yet to suggest that there is indeed plasticity in the olfactory bulbs of adult marsupials.

At P37 we observed a strong PrP^C^ immunoreactivity in the thalamus, a region which has a strong nonphotic influence on sleep and circadian rhythmicity [41]. This finding suggested an evolutionary conserved involvement of PrP^C^ in sleep homeostasis in the Op, in which a functioning circadian timing system exists [42]–[44].

Before weaning, PrP^C^ was detectable in the parenchyma of the hippocampus (Figure 3B). Interestingly, in different eutherian species, PrP^C^ preferentially localizes in specific hippocampal layers. In the adult Op (Figure 5C) and SHa [7] the strongest immunoreactive strata are the *oriens* and the *radiatum*, whereas MoPrP^C^ specifically localizes in the *stratum lacunosum-moleculare* (Figure 6A). These results seem to suggest a different regulatory role of PrP^C^ in the synaptic activity of different species. The lack of PrP^C^ in the nerve cell bodies was implied by the absence of signal in the pyramidal cell layer and granule cell layer of the dentate gyrus in both Mo and Op.

The most striking difference observed between the two species was the different localization of PrP^C^ in the white matter. The lower PrP^C^ signal in Op white matter structures argues for a lower expression of the protein by glial cells and neuronal axons. In P30 mice instead, a strong PrP^C^ immunoreactive signal was detectable in the *corpus callosum*, a specific eutherian structure enriched in myelinated axons and involved in interhemispheric communication [45].

### Implications for TSE pathology

The different ability of prions to infect certain species is apparently encoded by their structural features, which result in different physio-pathological outcomes [46]. Indeed some species may result resistant to prion infection. This strain-like behavior is known as the prion transmission barrier. However, under controlled laboratory conditions, prions are able to adapt and infect species previously believed to be TSE resistant, as was recently reported in rabbits infected by the murine ME7 prion strain using protein misfolding cyclic amplification (PMCA) techniques [47].

Structural studies on the recombinant PrP of mammals for which no TSEs have been reported in natural conditions [e.g. horse, rabbit and the marsupial Tammar wallaby (*Macropus eugenii*)], postulated that resistance to prions might be due to some structural features in the globular domain of those mammalian PrP sequences [48]–[50]. The OpPrP sequence presents an outstandingly large number of amino acid substitutions at the N-terminus in the copper binding sites and, within the C-terminus domain, in epitopes (residues 163–174 and 221–230 in Mo numbering, Figure 1) critical for prion conversion [22], [51]–[54]. Based on this sequence identity analysis, it is possible to argue that these amino acidic differences might have an impact on the ability of OpPrP to sustain prion conversion. On the other hand, if structural differences in mammalian PrP are important for understanding the molecular mechanisms of TSEs, the neuronal distribution of PrP^C^ in mammalian species that are putatively resistant to prion diseases should be considered.

It is noteworthy that PrP^Sc^ accumulates in the white matter areas of Mo and SHa brains, thus suggesting that glial cells may be the primary targets for prions [35], [55]. Indeed, the infectious agent has been shown to spread from the needle track along white matter pathways towards the gray matter [56]. This hypothesis is strengthened by pathological studies in human brains of terminal CJD patients showing axonal damage, hence suggesting a transport of prions through white matter pathways [57].

Although prion diseases have not been reported in the Op so far, the differential expression profile might account for a different susceptibility to prions in general or to diverse prion strains in particular, as well as for a different pattern of PrP^Sc^ accumulation and propagation between placentals and marsupials. To understand the biological and neurological significance of our observations, it would be of interest to attempt specific prion infectivity experiments in this mammalian model.
